# The Education of the Medical Profession by the War

**Published:** 1918-07

**Authors:** 


**A Monthly Review of Medicine and Surgery**

EDITOR AND PUBLISHER, DR. A. L. BENEDICT, 228 Summer St., corner of Elmwood Ave., Buffalo, (Address for all communications. Please make personal and telephone calls before 1 P. M.)

Third, in age, on the western continent. Independent, but supports professional organizations. News limited mainly to Western N. Y. and Penn.

Subscription $2.00 a year; $3.00 for two years in advance. Changes and errors in address or failure or duplication of delivery, should be reported immediately.

Yearly Volume 73 JULY, 1918 Number 12

### The Education of the Medical Profession by the War.

It is unnecessary to say that the War provides ample opportunity for acquiring experience along surgical lines. It may not be out of place, however, to call attention to several facts that may not be so apparent. This surgical experience is by no means limited to traumatisms that will be encountered rarely after peace is established. Indeed, a good deal of the technic of war surgery has been acquired and is being taught, not only didactically, but clinically in this country, based on traumatisms that closely simulate in their mechanic and bacterial results, those of war. For example, a compound fracture of the leg due to an automobile, with the limb ground into the mixture of dirt and manure on a city pavement, is practically identical with one due to a shell that caves the roof of a dug-out onto a soldier and covers the wound with Belgian soil. The large amount of material available for study and the free availability of bacteriologic examinations has already led to results showing that what have been considered non-specific bacteria are specific even so far as presumptive identification by clinical manifestations and preferential treatment by selected antiseptics are concerned. A point whose importance has scarcely been realized and which applies both to surgery and to internal medicine, is that military discipline, applied to the medical corps, tends to standardize methods of treatment, to eliminate personal peculiarities, and yet, in the long run, to determine the best method.

The experience in communicable diseases, in the control and cure of venereal diseases, in psychiatry and many other lines, is already of great value and this value will be greatly increased even in a year.

But there are educational factors at work broader than opportunities to acquire detailed experience. At no time in the history of the country have so many medical men, especially of the rank and file, been relieved of the immediate necessities of practice and given post-graduate courses of all kinds, from the rather superficial broadening teaching, didactic and clinical, which will vastly improve the imperfectly prepared or rusty general practitioner as well as the specialist who has deliberately neglected to keep himself informed on various matters which he needs even to make him a good specialist, up to technical courses of the highest order. To a large degree, these forms of post-graduate training have been lacking on account of financial reasons but, to perhaps to an equal degree, because teaching forces have been rendered available as a matter of patriotism which were formerly refused as being occupied with official duties or even for narrowly selfish reasons.

With the exception of the A. M. A. meetings and a few congresses of short duration and many distractions and without any power to inforce actual study, more physicians have been gathered together than at any other period. The absolute equality of men thus gathered for intensive training, so far as they themselves are concerned and their absolute dependence on and subjection to authority is unprecedented. The steam roller process of crushing egoism and pomposity and the substitution for prestige along pre-existing lines, of a nearly equal financial status and of opportunities for rank according to somewhat different lines of ability may be expected to produce a more democratic spirit, yet with due recognition of merit, wherever found.

Most of the civil population has had a full conception of the term soldiering or "sojering." Whatever justice this conception may have had in the past, a spirit of hard work now permeates the army and only one ignorant of military matters can criticise some part of the work as a waste of energy. To a large degree, the minor hardships of military life and especially those that seem to be due to arbitrary requirement of inconveniences, are intended to adjust a man to the inevitable hardships of active service. One not only learns to "live in his trunk" but to be clean and to do his work under conditions approached in civil life by the hour or so when packed for a train that will leave in less than an hour. Odds and ends of time, periods of a few minutes otherwise wasted, must be used for shaving and other details of the toilet, attending to correspondence, and for study. Recreation and longer tasks must be done in periods of a few hours formerly devoted to minor requirements. On active duty, the surgeon must attend to a number of patients considered impossible by most truthful physicians in civil life and to an amount of book keeping which the civilian either leaves to a trained assistant or neglects entirely.

